# Multiple Foreign bodies entrapped at Duodenal Web

**DOI:** 10.21699/ajcr.v8i3.578

**Published:** 2017-05-01

**Authors:** Aditya Pratap Singh, Ramesh Tanger, Dinesh Kumar Barolia, Arun Kumar Gupta, Sunil Kumar Mehra

**Affiliations:** Department of Pediatric Surgery, SMS Medical College Jaipur, Rajasthan, India.

**Dear Sir,**

Duodenal web with a small aperture usually presents late with bilious or non-bilious vomiting that exacerbates on starting proper weaning food. We report a case of duodenal web in a child where on exploration multiple foreign bodies (FBs) were found entrapped in the duodenum.

An 18-month-old male infant presented with intermittent non-bilious vomiting with pain abdomen for last six months. There was history of localized abdominal distension which gets relieved partially, with vomiting. Patient was born at full term and had no previous medical or surgical history. On examination, patient’s weight was 8.8 kg. Biochemical profile was normal. X-ray abdomen revealed gastric distension with paucity of gas in rest of abdomen. In upper GI study, stomach and duodenum were distended with narrowing of the 2rd part of the duodenum with passage of the contrast distally, suggestive of the perforated duodenal web (Fig.1). At laparotomy, duodenotomy revealed a web with a pin point hole in the center. Multiple FBs were present in the duodenum. The web was excised and the foreign bodies were retrieved (Fig.2). The foreign bodies were mostly buttons, seeds and diamonds. Postoperative period was uneventful.

**Figure:**
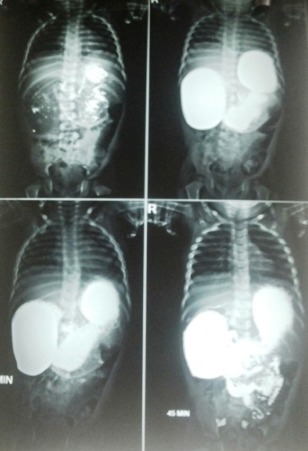
Figure 1: Showed contrast study indicative of partial duodenal obstruction.

**Figure:**
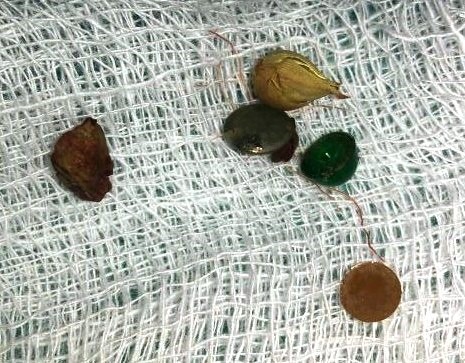
Figure 2: Showed retrieved foreign bodies.

Congenital duodenal obstructions might be complete or partial and can be classified as either intrinsic or extrinsic. The intrinsic lesions include primarily duodenal atresia or web.[1,2] Duodenal webs may present late with features of partial duodenal obstruction; delay in presentation depends upon size of the aperture of duodenal web. Bigger apertures may even delay its diagnosis until adulthood.[3] Vomiting may get exacerbated when weaning is introduced. In our case, the presentation was a bit delayed but entrapment of multiple FBs made it interesting.

## Footnotes

**Source of Support:** Nil

**Conflict of Interest:** None declared

